# *Raoultella planticola* Septicaemia in an Immunocompromised Patient—A Case Report

**DOI:** 10.1002/ccr3.70162

**Published:** 2025-02-26

**Authors:** Sanmithra Patavardhan Koppa Arunakumar, Karunamuni (Indika) Karunaratne, Robert Fassett

**Affiliations:** ^1^ Tasmanian Health Service—Northwest Region Burnie Tasmania Australia

**Keywords:** antibiotics, bloodstream infections, metastatic cancer, *Raoultella planticola*

## Abstract

Bacterial bloodstream infections, including peripherally‐inserted‐central‐venous‐catheter‐related‐bloodstream‐infection (PICCR‐BSI), are common in febrile neutropenic cancer patients. *Raoultella planticola* , a rare gram‐negative bacterium, can cause multi‐organ infections, especially in immunocompromised individuals. Early detection and appropriate antibiotic therapy improve outcomes. Careful assessment of clinical factors can help prevent PICCR‐BSI and enhance patient care in cancer settings.

## 1 Introduction

Bacterial bloodstream infections (BSI) account for approximately 20%–30% of all febrile neutropenic episodes in adults with malignancy [1]. There has been a shift in etiology of BSI from gram‐positive to gram‐negative organisms depending on the geographic area [2]. In cancer patients, the peripherally inserted central venous catheter related bloodstream infection (PICCR‐BSI) incidence rate is 2.6 per 1000 catheter days, 78% of which are mainly caused by gram‐negative bacteria and 43% are caused by gram‐positive bacteria [3]. *Raoultella planticola* ( *R. planticola* ) is a gram‐negative, aerobic, nonmotile bacterium that can be found in soil and water. Urine, feces, and sputum of 9%–18% of humans have *R. planticola* colonization. Although both immunocompetent and immunocompromised persons can develop *R. planticola* bacteraemia, 82.4% of patients are immunocompromised [4]. There have been multiple cases of urinary tract infections and a few reports of bacteraemia with *R. planticola* as the cause.

## 2 Case History and Examination

A 57‐year‐old female of European descent presented to our hospital with fevers and feeling unwell for 2 days prior to admission on a background of extensive metastatic stage (IV B) non‐small cell lung cancer. She was receiving palliative radiotherapy to brain, chest, and neck and had completed 6 cycles of carboplatin and etoposide chemotherapy. On examination, her temperature was 37.9°C, she had a Glasgow Coma Scale of 15/15, her vitals were stable. However, she was clinically dehydrated with loud upper airway sounds heard on chest auscultation. A single lumen PICC line was present in the left arm which was inserted in June 2022 for chemotherapy. Infection markers, biochemistry, and blood culture were sent.

## 3 Investigations, Differential Diagnoses and Treatment

Investigations revealed anemia (Hemoglobin: 87 g/L, Hematocrit: 0.26) and elevated white cell count (16.6/nL). The neutrophils showed left shift with moderate toxic changes (14.7/nL). C‐reactive protein (CRP) had raised to 394 mg/L (normal: < 5 mg/L). Biochemistry revealed features of acute kidney injury [estimated glomerular filtration rate (eGFR): 50 mL/min/1.73 m^2^ and serum creatinine: 106 μM/L]. Polymerase chain reaction test for Coronavirus disease‐19 (SARS‐CoV‐2) was negative. Chest X‐ray showed an ill‐defined confluent right upper lobe consolidation with coarse reticular markings. There was no evidence of pulmonary embolus on computerized tomography pulmonary angiography (CTPA). However, new metastatic masses were noted along with superior vena caval occlusion and enlarged mediastinal and hilar lymph nodes. Suspecting right sided pneumonia, piperacillin/tazobactam was started along with intravenous fluids and her regular medications.

She had a Medical Emergency Team (MET) call on the second day of admission for tachycardia (heart rate: 140 s) and hypothermia (temperature: 34°C). No other symptoms and signs were noted. Sinus tachycardia was observed on electrocardiogram. Other vital parameters were stable. No visible signs of infection were noted at the PICC line site. Blood investigations were repeated, and metoprolol was added. Initial blood culture from a peripheral intravenous cannula (PIVC) in the Emergency Department had not shown any growth (12/09/2022 at 2245 h). However, the subsequent blood culture during the MET call (14/09/2022 at 1740 h) from another new PIVC revealed *Stenotrophomonas maltophilia* and *R. planticola* (from anaerobic bottle), with both being sensitive to cotrimoxazole. Another sensitivity provided for *Stenotrophomonas maltophilia* was aztreonam. *R. planticola* was sensitive to ceftriaxone, gentamicin, amoxycillin‐clavulanate, cefotaxime, ceftazidime, piperacillin‐tazobactam, tobramycin, amikacin and aztreonam and resistant to ampicillin/amoxycillin.

The infectious disease (ID) team was consulted. Piperacillin/tazobactam was changed to oral cotrimoxazole. Repeat blood cultures were taken from the PICC line and a new PIVC site was ordered. Renal functions were monitored. Repeated blood culture report showed growth of *R. planticola* —from the PICC line (19/09/2022 at 1620 h) and was sensitive to amoxycillin‐clavulanate, ceftriaxone, gentamicin, piperacillin‐tazobactam, cefotaxime, ceftazidime, cotrimoxazole, meropenem, tobramycin, amikacin and aztreonam. This was resistant to ampicillin/amoxycillin. The PICC was removed on 20/09/2022 at 1245 h and the tip was sent for culture and sensitivity. The tip of PICC did not reveal any growth.

## 4 Outcome and Follow Up

The patient responded to treatment with cotrimoxazole. Clinically, she was improving apart from the cough with minimal sputum production. Inflammatory markers including CRP were trending downwards. Further blood cultures on 21/09/2022, 24/09/2022, and 25/09/2022 remained sterile after 2 days of incubation. The ID team advised to continue oral cotrimoxazole for 4 weeks.

Her overall condition declined gradually due to metastatic disease. The palliative care team was involved for end‐of‐life care. She passed away comfortably in hospital after 6 weeks of admission on account of metastatic disease.

## 5 Discussion

*R. planticola* is an aerobic, gram‐negative rod predominantly found in water and soil. It was previously known as *Klebsiella planticola* but in 2001, it was later reclassified in the new genus of *Raoultella*, partly due to its histamine‐producing properties. *R. planticola* is generally innocuous and rarely causes infection in humans [4]. The first reported infection was in 1986, but there has been an increasing number of associated reports since the year 2000 [5]. The incidence of *R. planticola* infection might have previously been underestimated due to the difficulty in isolating the bacterium and confusion with other bacteria, including Klebsiella spp.

*R. planticola* has been a cause for pneumonia, urinary tract infection, cholangitis, conjunctivitis, peritonitis, necrotizing fasciitis, bacteraemia, cellulitis, and soft tissue infection in both adult and pediatric populations. Shotaro Yamamoto et al. found 34 cases of *R. planticola* bacteraemia in their review of the literature in 2018. According to their findings, 70.6% of patients with *R. planticola* infection had a malignancy. Hematological malignancies and biliary tract neoplasms comprised 29.2%, pancreatic neoplasms were 16.7%, and others were 25.0%. They opined that the development of *R. planticola* bacteraemia was associated with an immunocompromised state, either due to the malignancy itself or because of the chemotherapy treatment [6].

*R. planticola* is sensitive to a wide range of antibiotics except Ampicillin. However, a few articles have suggested that the bacteria develop antibiotic resistance, especially with the carbapenems which have led to severe infections [7, 8]. The mechanism of its pathogenesis remains unclear in humans given the limited data availability. Immunocompromised state, proton pump inhibitor use, and chemotherapy increase the chances of infection [9]. *R. planticola* infection can occur when poorly cooked seafood is consumed in large quantities and affects various human organs, with no inclination toward a particular organ system [10].

PICC offers many advantages, but rising evidence points toward a higher risk of PICCR‐BSI, particularly in cancer patients undergoing chemotherapy or antibiotic therapy. Factors such as tumor type and number of catheter lumens increase this risk. Being mindful of these factors while assessing can help prevent infections and improve cancer patients' care [11].

Thus, we conclude by stating that it is prudent to be aware of *R. planticola* as a cause of bacteraemia in immunocompromised patients. The organism has been involved in infecting multiple organ systems. Individuals who are immunocompromised, with multiple comorbid diseases, including malignancy and stage 5 chronic kidney disease are at risk. *R. planticola* infection has a good prognosis if identified early and treated with the appropriate antibiotics.

## Author Contributions

**Sanmithra Patavardhan Koppa Arunakumar:** conceptualization, data curation, methodology, resources, software, writing – original draft. **Karunamuni (Indika) Karunaratne:** supervision, validation, visualization, writing – review and editing. **Robert Fassett:** conceptualization, formal analysis, supervision, validation, writing – review and editing.

## Consent

Signed consent from the family member (son) has been obtained.

## Conflicts of Interest

The authors declare no conflicts of interest.

## Permission to Reproduce Material From Other Sources

The authors provide the permission.

## Data Availability

Data was collected from the Digital Medical Record of the Tasmania Health Service.
